# Enhancing the Retention and Oxidative Stability of Volatile Flavors: A Novel Approach Utilizing O/W Pickering Emulsions Based on Agri-Food Byproducts and Spray-Drying

**DOI:** 10.3390/foods13091326

**Published:** 2024-04-26

**Authors:** César Burgos-Díaz, Fernando Leal-Calderon, Yohanna Mosi-Roa, Manuel Chacón-Fuentes, Karla Garrido-Miranda, Mauricio Opazo-Navarrete, Andrés Quiroz, Mariela Bustamante

**Affiliations:** 1 Agriaquaculture Nutritional Genomic Center, CGNA, Temuco 4780000, Chile; 2 Université de Bordeaux, CNRS, Bordeaux INP, CBMN, UMR 5248, 33600 Pessac, France; 3 Scientific and Technological Bioresource Nucleus (BIOREN-UFRO), Universidad de La Frontera, Temuco 4811230, Chile; 4 Laboratorio de Química Ecológica, Departamento de Ciencias Químicas y Recursos Naturales, Universidad de La Frontera, Temuco 4811230, Chile; 5 Centro de Investigación Biotecnológica Aplicada al Medio Ambiente (CIBAMA), Universidad de La Frontera, Temuco 4811230, Chile; 6 Department of Chemical Engineering and Centre for Biotechnology and Bioengineering (CeBiB), Universidad de La Frontera, Temuco 4811230, Chile

**Keywords:** Pickering emulsions, flavor retention, spray-drying, agri-food byproducts, D-limonene

## Abstract

Spray-drying is a commonly used method for producing powdered flavors, but the high temperatures involved often result in the loss of volatile molecules. To address this issue, our study focused on a novel approach: developing O/W Pickering emulsions with agri-food byproducts to encapsulate and protect D-limonene during spray-drying and storage. Emulsions formulated with lupin hull, lupin-byproduct (a water-insoluble protein–fiber byproduct derived from the production of lupin protein isolate), and camelina press-cake were subjected to spray-drying at 160 °C. The results revealed that these emulsions exhibited good stability against creaming. The characteristics of the dry emulsions (powders) were influenced by the concentration of byproducts. Quantitative analysis revealed that Pickering emulsions enhanced the retention of D-limonene during spray-drying, with the highest retention achieved using 3% lupin hull and 1% camelina press-cake. Notably, lupin-stabilized emulsions yielded powders with enhanced oxidative stability compared to those stabilized with camelina press-cake. Our findings highlight the potential of food-grade Pickering emulsions to improve the stability of volatile flavors during both processing and storage.

## 1. Introduction

Flavors are a variety of volatile organic compounds that play a significant role in human sensorial perception and affect the final acceptability of foodstuffs to the consumer [1,2]. However, most flavor compounds are highly volatile and unstable when exposed to external factors such as light, heat, and oxygen [3,4]. Therefore, to limit aroma degradation or loss during processing and storage, it is beneficial to encapsulate volatile flavors prior to use in foods or beverages. Among various methods for encapsulating volatile flavors, spray-drying stands out as the most widely employed technique due to its cost-effectiveness, high production capacity, and easy implementation [5]. By definition, spray-drying is the transformation of a feed in a fluid state (emulsion, dispersion, and solution) into a powder by spraying the feed into a hot drying gas [6]. The encapsulation process for volatile flavors involves three steps: emulsification of a core material, such as lipid–flavor, in a solution of a wall material, followed by atomization of the feed emulsion, and finally, the drying of the emulsion [7].

Before spray-drying lipophilic flavors, it is necessary to prepare an oil-in-water emulsion by dissolving the flavors in an edible oil and then emulsifying the mixture in a carrier solution using a homogenizer [8]. In this process, the properties of wall materials (carriers), the emulsion characteristics, and drying parameters are relevant factors that affect the encapsulation of flavors and the dehydration process [9]. Additionally, the stability of the emulsion is a crucial factor to take into account for flavor encapsulation, given that these compounds are typically water-insoluble [10]. According to Burgos-Díaz et al. [3], the retention of volatile compounds during spray-drying is closely related to the stability of the oil droplets of the O/W emulsion prior to the dehydration process. Therefore, numerous studies have been conducted to evaluate the retention of volatile flavors during spray-drying and shell life of the spray-dried powder [9,11,12,13]. Doi and coworkers [14] reported that it is possible to control the release profiles of volatile flavor through the emulsion structure and properties. Additionally, the same authors emphasized the importance of selecting an appropriate colloidal system to achieve a stable emulsion before spray-drying, thereby enhancing the retention and stability of volatile flavors. 

In this context, the use of particle-based emulsions known as Pickering emulsions has gained considerable attention due to their outstanding stability and functionality. In these colloidal systems, stabilization is accomplished by employing solely solid particles instead of surfactants or polymers [15]. Compared to the conventional surfactant-stabilized emulsions, Pickering emulsions have low toxicity, high stability against coalescence, and environmental friendliness [16]. Moreover, Pickering emulsions appear to offer significant advantages in the development of encapsulation and delivery systems for bioactive compounds compared to conventional emulsions [17]. In this context, recent studies have demonstrated the promising potential of Pickering emulsions for developing novel and effective controlled delivery systems for drugs and antioxidants [18,19]. Consequently, this type of colloidal system could serve as an effective alternative for producing powdered emulsions, addressing issues related to flavor instability during spray-drying and storage. It should be noted that studies related to this area remain practically unexplored.

The use of Pickering stabilizers derived from agri-food byproducts, such as oil seeds, fruits, and legumes, has attracted great interest due to their techno-functional properties and low cost [20]. Byproducts are a source of natural emulsifier molecules and amphiphilic particles, and this makes them good candidates to be used as Pickering stabilizers [21]. Furthermore, the food and pharmaceutical industries favor natural Pickering particles due to their notable natural benefits, including being a renewable resource, easy preparation, excellent biocompatibility, and unique interfacial properties. Consequently, this study faced the challenge of evaluating the functionality of various agri-food byproducts (lupin hulls, lupin-byproduct, and camelina press-cake) as potential Pickering stabilizers and wall materials for encapsulating flavor compounds through spray-drying. These agri-food byproducts were selected based on their chemical composition, which includes insoluble proteins, amphiphilic agents, dietary fibers, among others. These nutritional characteristics make them promising candidates for use as Pickering stabilizers.

Given the aforementioned considerations, this original study aims to explore the impact of the composition and structure of Pickering emulsions on their barrier properties, specifically their ability to encapsulate and protect volatile flavors within oil droplets. Notably, powdered O/W Pickering emulsions stabilized by agri-food byproducts have not been previously employed as a system for encapsulating volatile flavor compounds through spray-drying. In this research, D-limonene served as a model flavor due to its widespread use as a natural flavoring and fragrant agent in various industries such as food, cosmetics, perfume, pharmaceuticals, and cleaning products, owing to its pleasant lemon-like odor [5]. However, D-limonene is highly unstable, prone to volatilization in the presence of light, air, moisture, and high temperatures. Furthermore, it is extremely vulnerable to oxidation and chemical transformations, resulting in an unpleasant taste and odor [3,5]. These characteristics limit its practicality as an ingredient in industrial applications. Consequently, a primary objective for both researchers and industries is to safeguard D-limonene from harsh conditions, aiming to prevent or minimize volatile losses and deterioration during processing and storage. It is noteworthy that the development of emulsion-based encapsulation systems with appropriate flavor release profiles remains a challenge, given the multitude of factors influencing release and retention during storage.

## 2. Materials and Methods

### 2.1. Raw Materials

Agri-food byproducts based on lupin hull, lupin-byproduct (a water-insoluble protein–fiber byproduct derived from the production of lupin protein isolate); and camelina press-cake were provided by the Agriaquaculture Nutritional Genomic Center (CGNA), Temuco, Chile. The chemical composition of each byproduct is outlined in Table 1. 

### 2.2. Treatment of Agri-Food Byproducts as Pickering Stabilizers

The agri-food byproducts were treated according to Burgos-Díaz et al. [22] with some modifications. Before grinding, the lupin hull sample was manually cleaned to remove any remaining lupin grains; the lupin-byproduct was washed three times with distilled water to eliminate any residual soluble macronutrients, and the camelina press-cake powders were defatted with hexane. To reduce particle size, all samples were milled using a rotor mill (Fritsch Mill Pulverisette 14, Idar-Oberstein, Germany) and sieved through a 120 μm aperture mesh to obtain a fine powder as a Pickering stabilizer. 

### 2.3. Composition of Powder Agri-Food Byproducts

The protein content was determined by the Dumas method (Dumatherm^®^ N PRO analyzer, Königswinter, Germany) using a conversion factor of 6.25. The oil content was determined according to the AOAC official method 920.39 [23]. The total dietary fiber was determined by the enzymatic gravimetric method (AOAC Official Method 985.29) [23]. Moisture was determined by the oven method at 105 °C and measured using the gravimetric method (NCh 841 Of. 78). The carbohydrates (nitrogen-free extracts) were calculated by the difference. Results are expressed on a dry weight basis.

### 2.4. Determination of Soluble and Insoluble Fraction

To determine the soluble and insoluble fraction of each byproduct, 5% (*w*/*w*) of each byproduct sample (obtained from Section 2.2) was dispersed in deionized water and stirred for 20 min. After that, samples were centrifuged for 1 h at 10,000× *g*. The supernatant containing the soluble fraction and pellet (water-insoluble fraction) was collected, and the soluble and insoluble (%) of the samples were calculated by gravimetry.

### 2.5. Preparation of D-Limonene-Loaded Pickering Emulsions

The emulsions were prepared according to Burgos-Díaz et al. [22] with some modifications. Powdered agri-food byproduct dispersions at different concentrations (from 0.25 to 4%, *w*/*w*) were used as the aqueous phase of the O/W emulsions. The pH of the aqueous phase was fixed at 7 using a phosphate buffer solution (0.1 M). The oil phase of emulsions (10% *w*/*w*) was prepared by mixing 99.5 wt% sunflower oil with 0.5 wt% of D-limonene. A pre-emulsion was first obtained through high-speed homogenization, followed by microfluidization. Thus, each agri-food byproduct was dispersed in the buffered solution with a magnetic stirrer for 20 min, and then homogenized with a rotor–stator turbulent mixer (Kinematica, Polytron^©^ PT2500E, Luzern, Switzerland) operating at 6000 rpm for 2 min. The speed was then increased up to 8000 rpm, and the oil phase (containing D-limonene) was progressively added to the water phase (2 min). Stirring was prolonged for 10 min at 12,000 rpm. Then, the system was subjected to microfluidization and homogenized at a pressure of 600 bars using a high-pressure homogenizer (GEA Lab Homogenizer Panda PLUS 1000, Parma, Italy). The emulsion was submitted to 3 passes through the chamber. Finally, the obtained emulsions were stored at 4 °C after preparation.

#### 2.5.1. Microstructure Observation of Pickering Emulsions

The microstructure of Pickering emulsions was visualized using an optical microscope (Olympus-BX40, Tokyo, Japan) equipped with a camera to estimate the droplet size and aggregation state. An image of the sample was acquired using digital image processing software (Micro Video Instruments Inc., Avon, MA, USA). 

Confocal Laser Scanning Microscopy (CLSM) (Olympus Fluoview 1000, Tokyo, Japan) was used to assess the interfacial structure of Pickering emulsions. The protein fraction of each agri-food byproduct particle was dyed with rhodamine B before the emulsion preparation. The pictures of the samples were acquired using the FV-ASW (v. 1.7) software.

#### 2.5.2. Creaming Index of Emulsions

The emulsion stability against creaming of the emulsions was analyzed through the evolution of the creaming index (CI) for 45 days following the methodology described by Burgos-Díaz et al. [22]. Briefly, 5 mL of each O/W emulsion was deposited into a glass tube and then sealed to prevent water evaporation. CI percentage (%) values were determined by using Equation (1):

CI (%) = (H_s_/H_e_) × 100 (1)

where H_s_ corresponds to the serum layer height, and H_e_ is the total emulsion height.

#### 2.5.3. Spray-Drying of D-Limonene Pickering Emulsions

Before converting the liquid emulsions to powder, the samples were mixed with maltodextrin at different concentrations (as shown in Table 2) for increasing the total solid content in the emulsion. The agri-food byproduct particles and maltodextrin (at different ratios) were used as wall material in this study. The total concentration of solids (Pickering particles, maltodextrin, and oil phase) was 30% of the weight of the final emulsion. Maltodextrin was added to the previously prepared emulsions, and the solution was agitated for 1 h to ensure complete dissolution of the biopolymer. Then, the emulsions were dried by using a mini spray-dryer (Büchi B-290, Büchi Labortechnik AG, Flawil, Switzerland). The spray-dryer was operated at various inlet temperatures: 140 °C, 160 °C, and 180 °C, feeding rate: 5 mL/min, and aspiration rate: 85%. This screening revealed that drying at an inlet temperature of 140 °C resulted in low-quality powders with high humidity, whereas drying at an inlet temperature of 180 °C resulted in lower residual moisture, but it also showed a considerable reduction in D-limonene content, indicating the volatilization of this compound. Hence, in this study, we chose to utilize an inlet temperature of 160 °C. Finally, the obtained powders were collected and stored at room temperature until their analysis. 

### 2.6. Powder Characteristics

#### 2.6.1. Moisture Determination of Powders

The moisture content of food materials is a particularly important factor, since it determines the materials’ physical properties and the powder properties. The determination of moisture in the powders was based on the AOAC 93406 method [23]. A mass of 1 g of powder was dried using a forced air-drying oven (BOV-T50F, Fremont, CA, USA) at 105 °C for 24 h. Afterwards, the sample was weighed in an analytical balance (Sartorius Entris 224i-1S, Göttingen, Germany). The moisture content was estimated as the percentage by mass of the sample (1 g) using Equation (2):

(2) $$W(\%)=\left(\frac{M_{1}-M_{0}}{M_{1}-M_{2}}\right)\times 100$$

where M_0_ is the mass in grams of the melting pot, M_1_ is the mass in grams of the melting pot and the powder before drying, and M_2_ is the mass in grams of the melting pot and the powder after drying.

#### 2.6.2. Powder Dispersibility

Powder dispersibility was determined as follows: 200 mg of dry powder was dispersed in 10 mL of distilled water and stirred in a vortex mixer at 2000 rpm for 30 min. Subsequently, the dispersion was gently centrifuged at 760× *g* for 10 min in order to induce fast sedimentation of large aggregates. Then, a 9 mL aliquot of the supernatant was dried at 105 °C for 24 h. Finally, the water dispersibility was calculated using Equation (3):

(3) $$Dispersibility\left(\%\right)=\left[\frac{\left(wf\times \left(10/9\right)\right)}{wi}\right]\times 100$$

where *wf* and *wi* are the final and initial masses in grams, respectively.

#### 2.6.3. Morphological Characterization of Powdered Emulsions

The microstructural properties of powdered emulsions were observed with a scanning electron microscope (SEM) SU3500 (Hitachi, Japan) at 12 kV, 50 Pa vacuum, and Backscattered Electron signal (BSE). 

### 2.7. Quantification of Microencapsulated D-Limonene Using Gas Chromatography Coupled to Mass Spectrometry (GC-MS)

The total amount of D-limonene was measured according to Burgos-Díaz et al. [3] with some modifications. Briefly, 500 mg of the spray-dried powder was dispersed in 2 mL of water. Subsequently, 2 mL of hexane was added to the mixture, which was then vigorously vortexed for 5 min. The resulting mixture was heated at 85 °C for 30 min, and then the extracted mixture was centrifuged at 1800× *g* for 10 min. Finally, samples of each emulsion were analyzed by a gas chromatograph (GC-2030 Nexis; Shimadzu, Kyoto, Japan) equipped with a mass spectrometer detector. The capillary column was Rtx-5MS (30 m × 0.25 mm, 0.25 μm df; Restek GC Columns, Bellefonte, PA, USA). Helium was used as the carrier gas. The column temperature profile was as follows: 40 °C for 1 min, then increased to 280 °C at a rate of 20 °C/min and held at this temperature for 3 min. The injector and the interphase were performed at 280 °C, and the detector was held at 230 °C. The electron impact ionization energy was set at 75 eV. A total of 1 μL aliquot from all agri-food byproducts was injected into the GC-MS. The external standard method was used to calculate the D-limonene concentration. 

D-limonene retention during storage was tested by incubation of 25 g of the powder under constant temperature at 25 °C. Total D-limonene concentration was determined as previously described with GC-MS chromatography at the beginning (t = 0) and after 30 days (t = 30). Total D-limonene content was compared to total aroma content at t = 0.

### 2.8. Quantification of D-Limonene Released from Agri-Food Byproducts by HS-GC-FID

The quantification of D-limonene released from the three dry emulsions at a given temperature was carried out using a Headspace autosampler Turbomatrix 40 coupled to a Clarus 680 gas chromatograph with a flame ionization detector (FID) from Perkin Elmer, Waltham, MA, USA (HS-GC-FID). To this end, 500 mg of each dry emulsion was placed in a 22 mL vial and subjected to three different temperatures: 25 °C, 35 °C, and 65 °C, for a duration of 15 min. The released D-limonene was then analyzed by GC-FID using a BPX5 capillary column (30 m length × 0.25 μm film thickness × 0.25 mm, SGE Forte, Trajan Scientific, and Medical, Ringwood, VIC, Australia). The GC-FID operational conditions were as follows: the injector temperature was set at 250 °C, and the carrier gas used was helium at a flow rate of 2.0 mL/min. The oven temperature program consisted of an initial temperature of 40 °C for 0.5 min, followed by an increase to 250 °C at a rate of 9 °C/min, and then holding at 250 °C for 2 min. The detector temperature was set at 250 °C, with hydrogen gas flowing at a rate of 45.00 mL/min and air at a rate of 450.00 mL/min [24]. The concentration of D-limonene was determined by analyzing the peak area obtained from the GC-FID chromatogram.

### 2.9. Oxidative Stability of D-Limonene from Spray-Dried Powders

The oxidative stability of encapsulated D-limonene was determined according to Sultana et al. [2] whith some modifications. In brief, 1 g of recently prepared spray-dried powder, containing encapsulated D-limonene, was placed in closed Falcon tube and stored inside a desiccator at a constant temperature of 25 °C (in a drying oven) for 6 months. All samples were incubated without adding water to the powder. Limonene oxide was chosen as the oxidative derivative of D-limonene. The extraction procedure and quantification of oxide limonene was the same as the procedure mentioned in Section 2.7.

### 2.10. Encapsulation Efficiency of Limonene (EE)

The encapsulation efficiency was determined by quantifying the total and surface D-limonene from the powders. To determine the not-encapsulated D-limonene of powders, 500 mg of powder was mixed with 15 mL of hexane and shaken slowly for 30 s. Then, the mixture was filtered through Whatman No. 1 filter paper, and the filtered powder was washed 3 times with 20 mL of hexane. To determine the total D-limonene content of powders, 500 mg of the powder was dispersed in 2 mL of distilled water in a glass tube. Then, 2 mL of hexane was added to recover D-limonene from the solution. The total solution was mixed and vortexed vigorously for 5 min. To extract the aroma from powder to the organic solvent, the mixture was heated at 85 °C for 30 min. The extracted mixture was then centrifuged at 3000 rpm for 10 min to separate the organic phase from the water. Afterwards, samples were analyzed by gas chromatography (GC-2030 Nexis; Shimadzu, Kyoto, Japan). EE was calculated from Equation (4):

(4) $$EE(\%)=\left(\frac{Total\;limonene-Surface\;limonene}{Total\;limonene}\right)\times 100$$

### 2.11. Statistical Analysis

The statistical software Statistix 10 (Tallahassee, FL, USA) was used to perform the analysis. An analysis of variance (ANOVA) followed by Tukey’s test was carried out to assess the effect of Pickering stabilizer treatments on D-limonene retention, moisture, dispersibility, encapsulation efficiency, and creaming index of the powder. On the other hand, a t-test was performed to analyze differences between 0 and 30 days for D-limonene retention. All data were assessed for normality and homoscedasticity of variance. Values of *p* ≤ 0.05 were considered significant. Finally, results are expressed as means and their corresponding standard errors.

## 3. Results

### 3.1. O/W Pickering Emulsions Characterization Prior to the Spray-Drying Process

**Figure 1:**
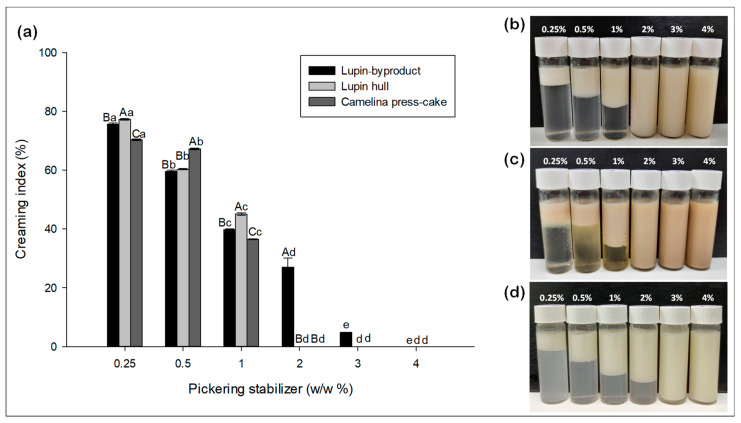
The graph corresponds to the creaming index of Pickering emulsions (**a**); Visual observation of O/W Pickering emulsions prior to spray-drying stabilized by lupin hull (**b**); camelina press-cake (**c**); and lupin-byproduct (**d**). Different capital letters indicate statistically significant differences (*p* ≤ 0.05) among individual concentrations of Pickering emulsions, whereas different lowercase letters indicate statistically significant differences (*p* ≤ 0.05) among all concentrations of Pickering emulsions.

The emulsion stability was evaluated by the creaming index and visual observation of Pickering emulsions (Figure 1). Creaming occurs when the emulsion separates due to a density difference, where the lighter oil droplets rise to the surface. Thus, the destabilization caused by creaming leads to the separation of the emulsion into distinct phases, resulting in the formation of a transparent layer at the bottom of the sample tube, which is commonly referred to as clarification [22]. 

Figure 1a illustrates that emulsions stabilized by lupin hull and camelina press-cake at a concentration exceeding 2.0% (*w*/*w*) exhibited no creaming behavior after 24 h. For lupin-byproduct, the absence of creaming after 24 h was observed with a higher concentration of particles (≥3.0%, *w*/*w*). These observations suggest that the emulsion stability can be improved by gradually increasing the concentration of particles. Furthermore, as visually depicted in Figure 1b–d, there were no apparent signs of macroscopic oil leakage at the top of the tubes. 

Additionally, we observed that the viscosity of the obtained emulsions increased with the particle concentration, which aligns with previous reports [25]. Moreover, as noted by Joseph et al. [25], particles can bridge multiple droplets, thereby enhancing the connectivity of the medium. Consequently, across all three types of particulate stabilizers, we observed a consistent qualitative trend: creaming became less prominent, eventually becoming imperceptible as the particle content increased.

These stabilizers induced high viscosity in the emulsions due to their robust aggregated state, naturally delaying gravity-driven phenomena. For example, camelina press-cake contains a significant proportion of proteinaceous amphiphilic insoluble particles, which may promote the bridging flocculation of the emulsion droplets, thus increasing the average emulsion viscosity.

On the other hand, particles (insoluble fraction) tend to sediment when they are alone in solution because they are denser than the aqueous phase. Interestingly, Figure 1b–d show that when a “subnatant phase” forms at low particle concentrations, it is not only almost clear but also devoid of sediment. Therefore, it can be concluded that the particles are attached to the droplets as they rise with them during creaming.

**Figure 2:**
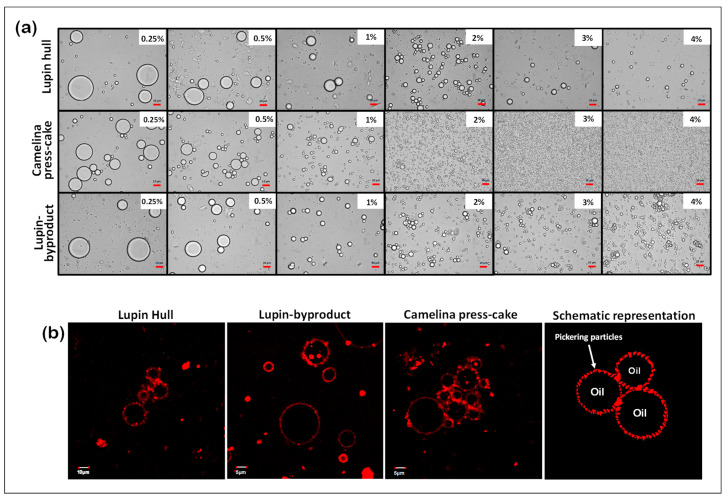
(**a**) Light microscopy images of emulsions stabilized with different Pickering stabilizer concentrations and prior to spray-drying; (**b**) CSLM images of emulsions stabilized by lupin hull, lupin-byproduct, and camelina press-cake.

Figure 2a shows micrographs of the emulsions prior to spray-drying. Interestingly, the micrographs did not reveal significant differences in the evolution of the droplet sizes with particle concentration among the different types of agri-food byproducts. The images show that the droplet size of the O/W emulsions decreased as the agri-food particle concentrations increased, from 0.25 to 4% (*w*/*w*) at a fixed oil phase concentration (10%, *w*/*w*). Joseph et al. [25] observed a similar qualitative trend in Pickering emulsions stabilized by plant particles. When the particle concentration rises at a constant droplet fraction, a larger interfacial area is covered, resulting in smaller droplets.

Figure 2a also reveals that the emulsion stabilized with camelina press-cake exhibited a smaller droplet size compared to those stabilized with lupin byproducts (at a concentration of ≥2.0% *w*/*w*), potentially influenced by its protein content (44.54% *w*/*w*, Table 1). According to Burgos-Díaz et al. [22], proteins are considered the primary surface-active compounds responsible for Pickering emulsion stabilization.

Representative CLSM images of particle-stabilized emulsions are shown in Figure 2b. The particles are clearly distinguishable, with the larger ones reaching sizes on the order of a few micrometers. When these particles are anchored at the interfaces, they form a thick layer generating a mechanical barrier against droplets coalescence. The thickness of this layer is typically much greater than the range of electrostatic or steric repulsions generated by conventional amphiphilic molecules such as surfactants and proteins. Such thick and rigid layer actually accounts for the outstanding stability provided by particulate stabilizers. The size of the particles and their concentration are consequently key parameters to monitor the droplet size in Pickering emulsions [26]. 

The observation of the micrographs in Figure 2b indicates that the oil droplets are effectively coated with a layer of the agri-food byproduct stabilizers, which is a clear confirmation that O/W Pickering emulsions have been obtained [22,27]. Furthermore, the presence of droplet bridging is clearly observable in Figure 2b, where particles are simultaneously adsorbed at the surfaces of two adjacent droplets. This phenomenon occurs when the number of particles is not sufficient to fully cover the oil–water interface, leading neighboring droplets to share the same particle. Likewise, this phenomenon can occur when particles are relatively large so that they can attach two or more oil droplets on their periphery. According to Schröder et al. [28], the bridging phenomenon in O/W emulsions happens when particles have dual wettability, even though they are predominantly hydrophilic. This type of bridging setup promotes the creation of large droplet flocs. These bridges play a role in preventing droplet coalescence, improving emulsion stability by effectively gelling the systems and slowing down creaming processes.

### 3.2. Powdered O/W Pickering Emulsions Characterization

#### 3.2.1. Effect of the Concentration and Type of Agri-Food Byproduct on Powder Characteristics

The visual inspection of the powders after the spray-drying process reveals an evident impact of the Pickering stabilizer content on the characteristics of the powders. Emulsions with a lower particle concentration produced larger, coarse powders, while emulsions with a higher particle concentration resulted in finer powders after the spray-drying (Figure S1, Supplementary Data). The use of Pickering stabilizers from agri-food byproducts enhances the quality of powders, making them well suited for producing powdered flavor in the food industry. This improvement aligns with the observed reduction in oil droplet size in the emulsions. Smaller droplets contribute to powders with better dispersibility and reconstitution properties, which are desirable when using the spray-drying process [29]. Similar findings were noted by Burgos-Díaz et al. [17], who found that emulsions stabilized with lower concentrations of lupin protein aggregates (LP-APs) produced coarse powders, while higher concentrations resulted in finer powders after spray-drying. 

Regarding the moisture content of the powders, the type and concentration of Pickering stabilizers did not show a significant impact or a clear trend (Table 3). The moisture values ranged between 3.17% and 4.47% for all powders. These slight variations can be attributed to the fact that certain macronutrients present in agri-food byproduct tend to retain more water during the drying process. Additionally, it is worth noting that all moisture content values stayed below 5%, which is deemed acceptable for a powdered food product, as per the guidelines recommended by CODEX standards [30].

In terms of dispersibility, it was observed that the values were relatively high (around 90%) for all powders (Table 3). No relationship was observed between the dispersibility and the concentration of each agri-food byproduct. For instance, all values remained between 88 and 91%, which contrasts somewhat with the reported “insoluble fraction” of each agri-food byproduct (lupin hull: 95.33%; lupin-byproduct: 95.55%, and camelina press-cake: 76.42%, Table 1). Indeed, increasing byproduct concentration, especially for its insoluble fraction, generally might lead to decreased dispersibility. Additionally, powder solubility often correlates with the type of wall material [31]. In this study, the dispersibility of powders may be influenced by various physicochemical properties, including chemical composition (protein, lipid, fiber, etc.), and physical parameters such as the particle size distribution and shape. Furthermore, the reconstitution behavior can be affected by factors like temperature, stirring conditions, mineral content, among others [32].

Table 3 also shows the encapsulation efficiency (EE) of the D-limonene contained in the powders. The results show that the EE of the microcapsules first increases significantly (*p* ≤ 0.05) with the amount of agri-food byproduct used to stabilize the emulsions prior to spray-drying. However, at a specific concentration of lupin hulls (>3.0% *w*/*w*) and camelina press-cake (>1.0% *w*/*w*), the EE diminishes by 20% and 26%, respectively. The outcomes exhibit a parallel trend to the investigation examining the influence of concentration on D-limonene retention during spray-drying (Section 3.3). Specifically, elevated concentrations of agri-food Pickering stabilizers correlate with a reduction in the D-limonene content within the resultant powders. On the contrary, the highest EE values obtained (around 90%) at a specific concentration of the agri-food byproduct could indicate the formation of an effective protective “shell” during the drying process, which confers a high level of protection to the encapsulated core materials [17]. In our study, the observed pattern in the EE, with an initial increase until reaching an optimum followed by a decrease, could be directly influenced by the particle concentration and/or viscosity of Pickering emulsions. Jafari et al. [9] suggest that there exists an optimum solids concentration for the spray-drying of volatile flavors. In some cases, adding more wall materials exceeds their solubility or dispersibility, and these undissolved wall materials cannot effectively encapsulate, leading to poorer flavor retention during the spray-drying. The same authors also suggest that increasing the solids concentration in the initial emulsion is advantageous until reaching a critical point for optimal viscosity. Beyond this point, increasing viscosity reduces the retention of bioactive compounds due to heightened exposure during atomization and difficulties in droplet formation during spray-drying. Furthermore, it has been demonstrated that a feed with higher viscosity leads to larger droplet sizes, and irregular particles are generated due to difficulties in droplet formation at elevated viscosities [9].

#### 3.2.2. Powder Morphology

**Figure 3:**
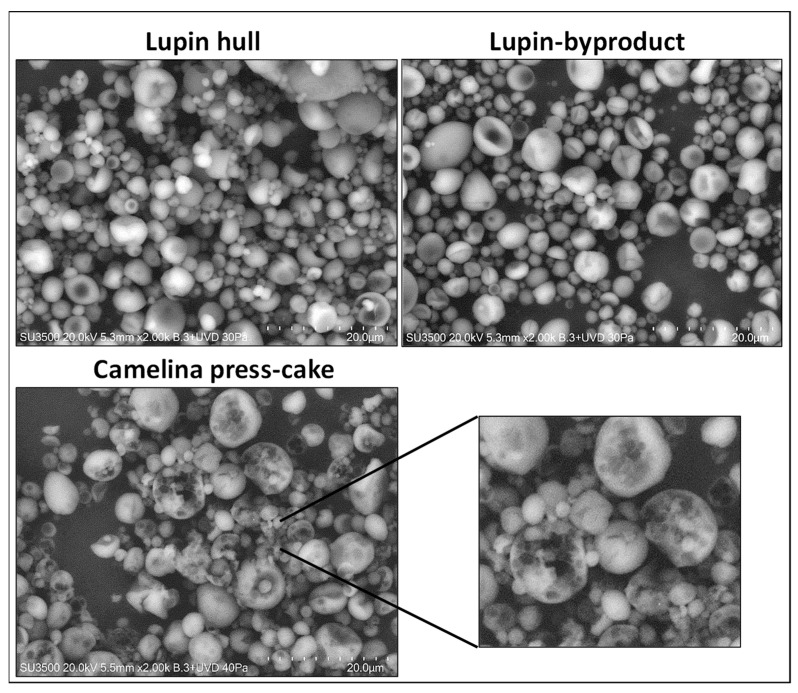
SEM images of the microcapsules obtained from the powdered Pickering emulsions.

Scanning Electron Microscopy (SEM) micrographs of the dry Pickering emulsions are shown in Figure 3. For spray-dried emulsions containing lupin hull and lupin-byproduct, the particles presented different sizes and irregular shapes with no visible cracks or fissures. This may indicate that microcapsules could reduce permeability to gases, thereby enhancing the protection and retention of encapsulated D-limonene. The occurrence of various sizes and “dentations” in microcapsules is a characteristic phenomenon frequently identified in particles produced via the spray-drying process. As per Carneiro et al. [33], the imperfections (teeth) are formed when there is a slow process of film formation during drying of the atomized droplets, associating the presence of surface depressions to the collapse suffered by the droplets during the initial stages of drying. The morphology of powder emulsions can be influenced by various factors, including homogenization conditions, the type of wall material, core material, atomizer conditions, among others [2].

The differences in the powder microstructure were more evident when comparing the images from the camelina press-cake with the other agri-food byproducts, since powders with lupin byproducts resulted in microcapsules with a smoother surface and fewer irregularities or rough areas. The powders derived from camelina press-cake exhibited visible wall cracks and a porous “sponge-like” microstructure (dark spots on the surface of the microcapsules, Figure 3), which could potentially promote the degradation of D-limonene through oxygen penetration and the release of volatile compounds. These findings align with the observed decrease in D-limonene retention at higher concentrations of camelina press-cake, as developed in the next section. A similar powder microstructure (sponge-like) was observed by Sarkar et al. [34] in spray-dried emulsions with ultra-high oil content.

### 3.3. The Effect of the Type of Agri-Food Byproduct and Concentration on D-Limonene Retention after Spray-Drying and Storage

**Figure 4:**
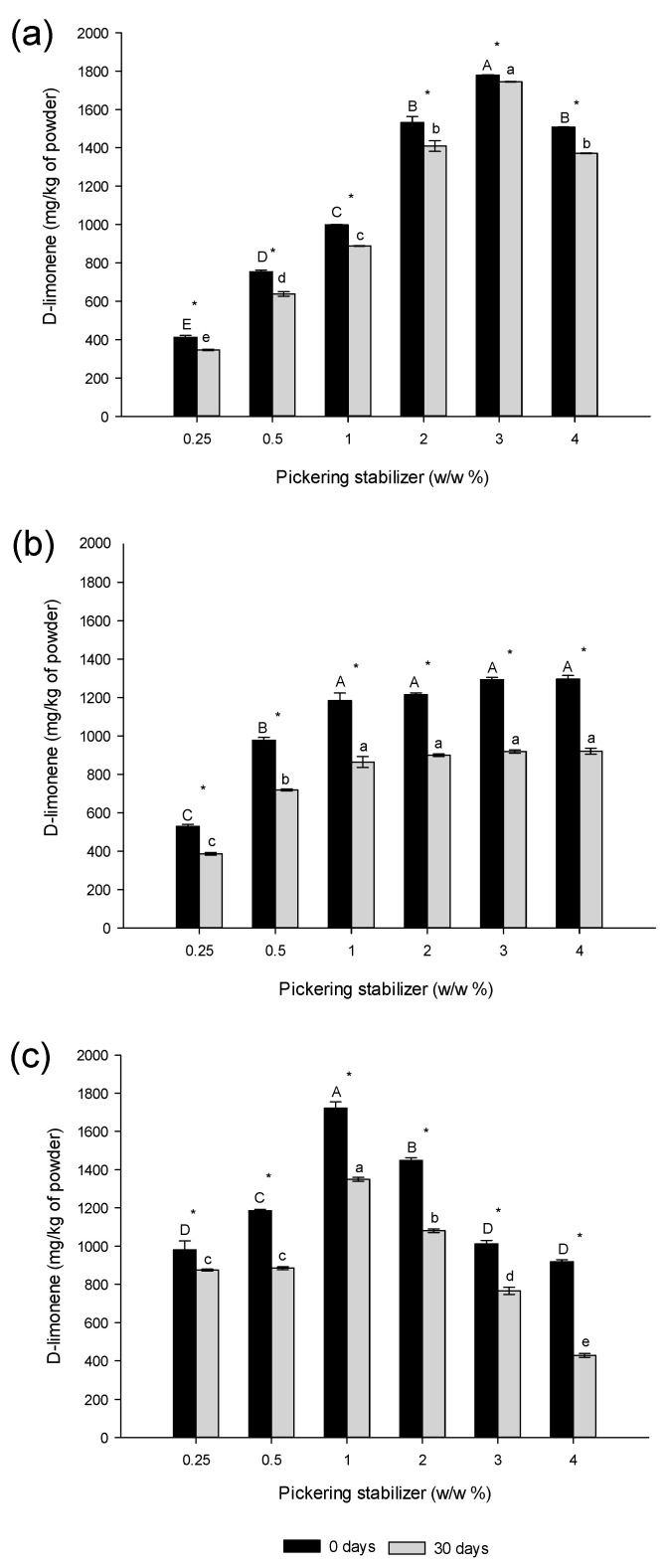
Effect of the type of agri-food byproduct on D-limonene retention right after spray-drying and storage for 30 days; (**a**) lupin hull, (**b**) lupin-byproduct, (**c**) camelina press-cake. Different capital letters indicate statistically significant differences (*p* ≤ 0.05) among all concentrations of Pickering emulsions at 0 days, whereas different lowercase letters indicate statistically significant differences (*p* ≤ 0.05) at 30 days. * indicates significant differences (*p* ≤ 0.05) between the days for each concentration of D-limonene.

Given the emulsion compositions outlined in Table 2 and under the assumption that only water is removed during spray-drying, the final D-limonene concentration would approximate 6700 mg/kg of powder. Figure 4 shows the evolution of D-limonene concentration in the powders as a function of the amount of Pickering stabilizer, for the different sources, both immediately after spray-drying and after a 30-day storage period. The values are below 30% of the expected theoretical value assuming zero loss of flavor. This outcome is not surprising given the high volatility of the molecule and the harsh drying conditions, including thermalization with a hot air stream at 160 °C. The flavor concentration in the powder could be enhanced by initially incorporating an overdose to account for potential losses.

Figure 4a shows that D-limonene concentration in the spray-dried powder varied from 412.5 to 1779.7 mg/kg of powder (*p* ≤ 0.05) as the lupin hull content increased from 0.25% to 3% (*w*/*w*). This represents a significant, nearly 4-fold increase. Surprisingly, the evolution was not monotonous: the spray-dried powder stabilized with 4% lupin hull showed an almost 20% reduction in the amount of D-limonene retained compared to the one formulated with 3%. A similar trend was observed within the same stabilizer concentration range after a storage period of 30 days. For lupin-byproduct, after an important increase in the concentration of D-limonene, no further significant evolution was observed at byproduct concentrations above 1%, at both t = 0 and t = 30 days (Figure 4b). Finally, in the case of camelina press-cake, the trend mirrors that observed with lupin hull, wherein the concentration of D-limonene initially rises before declining with the increase in byproduct content. Specifically, the peak retention of D-limonene is achieved at a 1% camelina press-cake concentration.

The fluctuating pattern of D-limonene retention could suggest the involvement of two opposing factors. On one hand, Pickering stabilizers are absorbed at the oil–water interface and also present in excess in the aqueous phase of the emulsions. Therefore, during and post the spray-drying process, they create a protective shell around the oil droplets, slowing down the diffusion of volatile flavors like D-limonene. As the proportion of Pickering stabilizer rises, the shells that surround emulsion droplets thicken, enhancing retention capabilities. Conversely, an increase in Pickering stabilizer content results in the formation of smaller oil droplets (as illustrated in Figure 2), thereby expanding the total interfacial area or, equivalently, the surface-to-volume ratio, likely hastening the outwards diffusion of D-limonene. This could account for the decrease in D-limonene retention observed at higher stabilizer concentrations. In addition to the effects previously discussed, it is important to consider other factors. The quality of the powder and the efficiency of encapsulation in the spray-drying process are influenced by various factors, including droplet size distribution, inlet temperature, feed flow rate, and drying air flow rate [35]. According to Jafari et al. [9], emulsions with smaller droplet sizes generally exhibit greater stability during the spray-drying process, which is crucial for achieving high encapsulation efficiency. Therefore, achieving smaller droplet sizes in the emulsion before the spray-drying process could enhance the content of encapsulated core material in the powders [35,36,37]. To achieve the expected results, the emulsion should be stable through the whole process of microencapsulation of the bioactive compound including short-term storage before the process, as well as transportation to the spray disc and the whole drying time [38].

On the other hand, the reduction in D-limonene concentration observed in the spray-dried powder stabilized with camelina press-cake and lupin hull could also be due to the increase in emulsion viscosity (from 3.0 cP to 7.5 cP approximately) prior to spray-drying, as the Pickering stabilizer concentration increased. This increase in viscosity can directly impact the characteristics of the powder. In our case, a fraction of oil containing D-limonene may have been lost during the spray-drying at high Pickering stabilizer percentages. This is in concordance with the viscosity values observed with the Pickering emulsions stabilized by camelina press-cake (7.86 cP) at 4%, which produced microcapsules with porosity and cracks or ruptures (as previously described in Figure 3). According to Janiszewska et al. [7], the emulsion stability and viscosity are relevant parameters for an effective microencapsulation process by spray-drying. An optimal viscosity of the emulsion could be achieved, depending on the type and characteristics of the spraying device. For example, a low viscosity of the aqueous phase facilitates the diffusion of essential oil droplets, potentially reducing the efficiency of microencapsulation [7]. Conversely, emulsions with high viscosity can clog the spray disc and produce larger nebulized droplets, which often are not adequately dried, leading to subpar atomization performance and powder characteristics [39,40]. 

### 3.4. Effect of the Temperature on D-Limonene Release from Powdered Pickering Emulsions

**Figure 5:**
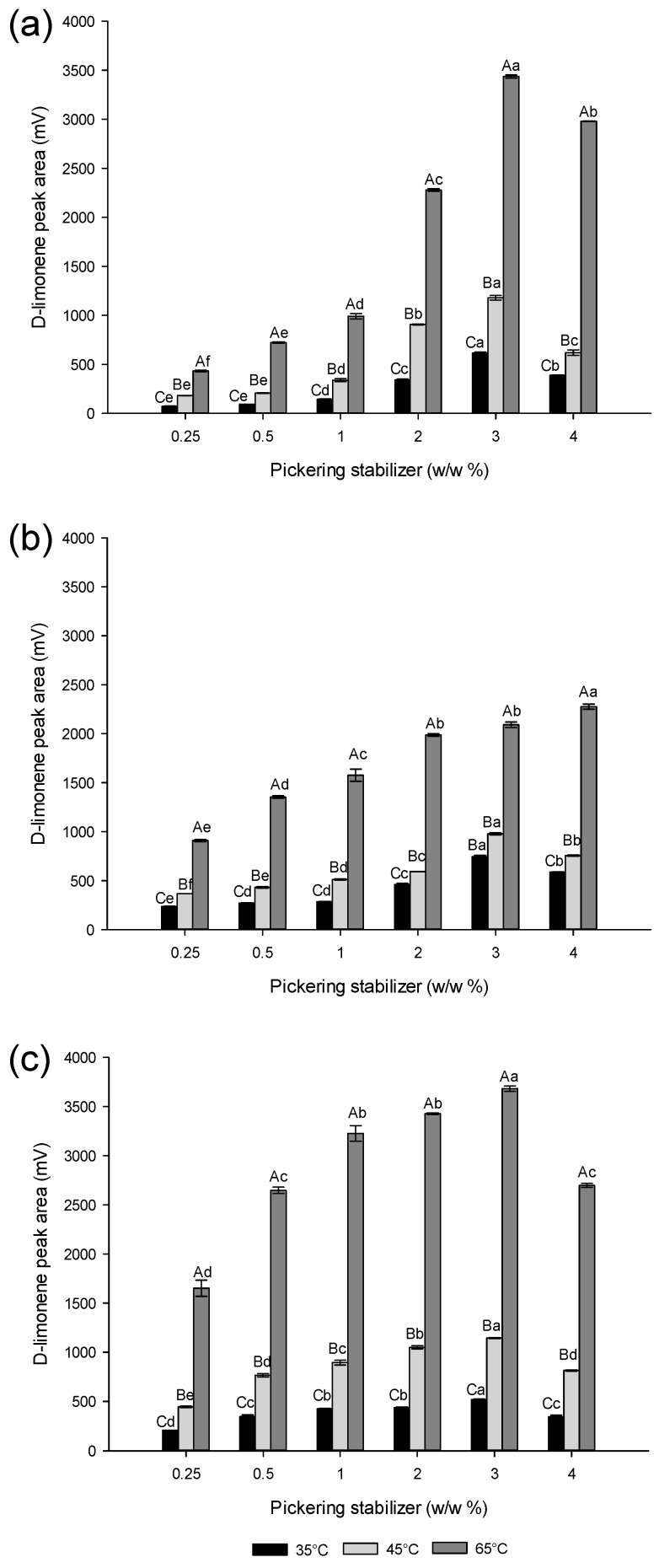
Effect of temperature on D-limonene release from powdered Pickering emulsions. (**a**) lupin hull, (**b**) lupin-byproduct, (**c**) camelina press-cake. Different capital letters indicate statistically significant differences (*p* ≤ 0.05) among individual concentrations of Pickering stabilizers, whereas different lowercase letters indicate statistically significant differences (*p* ≤ 0.05) among all concentrations of Pickering stabilizers.

Three temperatures (35 °C, 45 °C, and 65 °C) were used to promote flavor release from the powders in the headspace analysis. Under these conditions, the amount released was necessarily influenced by the quantity of flavor initially present in the sample. Thus, at the three explored temperatures, the behavior of the curves as a function of the Pickering stabilizer content in Figure 5 are qualitatively similar to those in Figure 4.

Indeed, dry emulsions based on lupin hull and camelina press-cake exhibit a non-monotonic variation passing through a maximum, while those based on lupin-byproduct show a monotonic growth tending to diminish at high Pickering stabilizer contents. As expected, the results indicated an increased release of D-limonene as temperature increased in all spray-dried emulsions and across different concentrations of Pickering stabilizers. However, variations in the release of D-limonene were observed depending on the agri-food byproduct source used to stabilize the emulsion before spray-drying. In general, dry emulsions containing camelina press-cake exhibited a greater release of flavor at all temperatures and Pickering stabilizer, compared to those formulated with lupin hull and lupin-byproduct. 

Additionally, Figure 5 shows absolute quantities (mV) of released D-limonene. However, a comparison of the relative delivery (i.e., normalized by the initial amount of D-limonene encapsulated) would be even more informative. This is possible indirectly by comparing the data from Figure 4 and Figure 5 with the camelina press-cake as a Pickering stabilizer. For example, at higher concentrations of camelina press-cake (higher than 1% stabilizer), the powder emulsions based on camelina press-cake showed a decrease in the limonene content at t = 0, from 1800 to 1000 mg/kg of powder approximately (Figure 4). However, the amount of D-limonene released from the same powder was highest at 65 °C at the same concentrations, equivalent to a signal of around 3500 mV (Figure 5c), despite the reduced amount of D-limonene present in the powder (Figure 4). Therefore, this powder stands out due to its significantly higher relative leakage rate compared to the others.

The increased release of D-limonene noted in spray-dried emulsions containing camelina press-cake, as shown in Figure 5c, can be ascribed to the microstructure of the powder. Indeed, the microcapsules displayed visible fissures and porosity (see Figure 3), potentially leading to an expedited release of D-limonene from the powder. Similar findings were reported by Takashige et al. [41], who observed a significant impact of wall materials, such as maltodextrin, lactose, and sucrose, on the release rate of D-limonene from spray-dried powder. Additionally, Soottitantawat et al. [42] established a correlation between flavor release rate and the glass transition temperature as well as the collapse temperature of wall materials. Similarly, Peng et al. [43] highlighted the dependency of flavor release from spray-dried powders on various factors including carrier material, emulsion characteristics, and powder morphology. Thus, the potential collapse behavior of wall materials should be taken into consideration when selecting them for flavor encapsulation.

### 3.5. Oxidative Stability of D-Limonene from the Spray-Dried Powders

**Figure 6:**
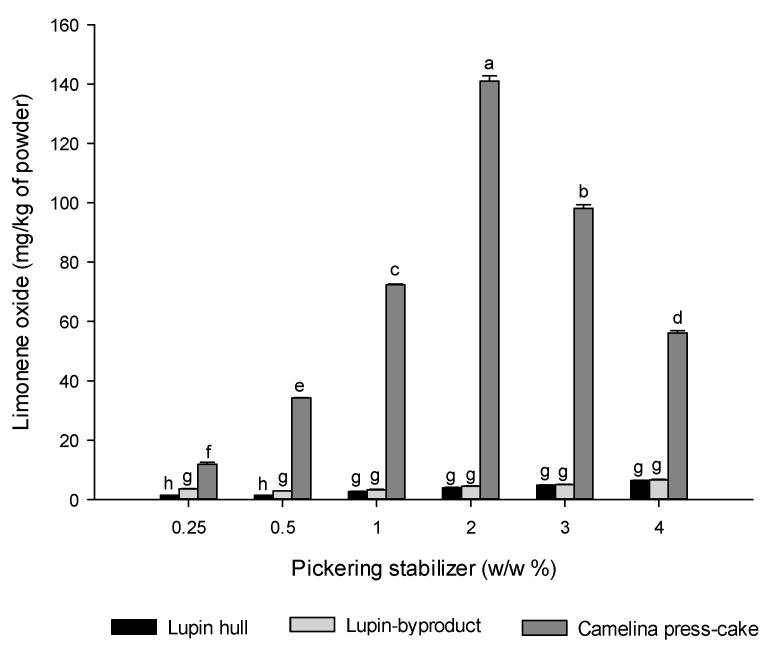
Oxidative stability of D-limonene for powdered Pickering emulsions. Different letters indicate statistically significant differences (*p* ≤ 0.05) among all concentrations of Pickering stabilizers.

All the spray-dried powders were evaluated for the oxidative stability of D-limonene during 6 months of storage. Limonene oxide (limonene-1, 2-epoxide) was chosen as an indicator of limonene oxidation due to this compound being one of its main oxidation derivatives [43]. The experimental results are reported in Figure 6. It is worth noting that at the beginning of the study (day 0), none of the samples exhibited oxidation products, indicating that the drying process did not lead to limonene degradation (data not reported in the graph). However, after six months of storage, the dry samples displayed the production of limonene oxide. The generation of limonene oxide significantly varied depending on the type of Pickering stabilizer. For powders derived from camelina press-cake, there was a notable increase in limonene oxide content (*p* < 0.05) after six months of storage compared to those from lupin hull and lupin-byproduct, suggesting that byproducts from lupin were more effective in preventing D-limonene oxidation during storage. For example, the limonene oxide content was 141.01 mg/kg powder in camelina press-cake, considerably higher than those observed with lupin hull and lupin -byproduct (6.42 mg/kg and 6.65 mg/kg, respectively). 

Regarding powders prepared with camelina press-cake, the formation of limonene oxide increased from 11.91 to 141.01 mg/kg powder at 0.25% and 2% (*w*/*w*), respectively. However, the formation of limonene oxide decreased at higher concentrations. The reduction in limonene oxide at concentrations exceeding 2% of camelina press-cake is possibly due to the decrease in the total limonene amount at higher concentrations of camelina press-cake, as described in Section 3.3. 

Haas et al. [44] suggest that the morphology and microstructure of the produced powder particles are the main factors governing the oxidative stability of labile compounds within the powder system, thus facilitating the oxidative stability of these compounds within the powder structure. In line with the aforementioned, the porous microstructure of the powders derived from camelina press-cake can influence oxygen permeability or the rapid release of D-limonene to the surface, thereby facilitating the oxidation of D-limonene in dry powders during storage. Studies on the microencapsulation of flavors and other bioactive ingredients using drying techniques have shown that structural characteristics, such as particle size distribution or internal porosity of the powder, affect oxidative stability during storage [44]. For example, the porous structure created by the drying process has been shown to provide little protection for sensitive components, leading to rapid oxidation during storage. 

In our case, the lower protective efficacy of spray-dried powders using camelina press-cake can be again attributed to the visible fissures and porosity of the microparticles, which enhance oxygen mobility. Microparticles from lupin hull and lupin byproducts exhibited lower limonene oxide formation, indicating that these byproducts offer a greater barrier to oxygen compared to powders stabilized by camelina press-cake. 

## 4. Conclusions

This study demonstrated that agri-food byproducts (camelina press-cake, lupin hull, and lupin byproduct) can be used to develop successfully solid-stabilized edible emulsions. In addition, O/W Pickering emulsions based on agri-food byproducts demonstrated to be an effective encapsulation system for D-limonene. Indeed, the study revealed that spray-drying can be used to transform the emulsions, prepared from blends of agri-food byproducts and maltodextrin as wall materials, into powders with protecting properties. The retention of D-limonene during spray-drying, the release of D-limonene due to temperature effects, and the oxidative stability of D-limonene during storage were significantly influenced by the type and concentration of Pickering stabilizers. Emulsions prepared with lupin byproducts resulted in powders with enhanced oxidative stability and powder characteristics compared with those prepared with camelina press-cake. The blends with 3% of lupin hull and 17% maltodextrin, and 1% of camelina press-cake and 19% maltodextrin, had the best performances in terms of protection of the D-limonene during the spray-drying process. The results also demonstrated that powders formulated with lupin byproducts were suitable wall materials to control the flavor release from spray-dried powder. 

Therefore, when it comes to flavor encapsulation, the key consideration lies in choosing the appropriate wall material to preserve the flavor during spray-drying and regulate its release from the resulting powder. Utilizing a blend of agri-food byproducts and maltodextrin presents a promising alternative for creating a suitable wall material for microencapsulation of volatile flavor compounds. This combination offers relatively good oxidative stability and protection during processing and storage. Additionally, encapsulating volatile flavor compounds in a Pickering powder that can be easily dispersed in water is likely to broaden its potential applications in the food industry, given that most food products contain a water-based phase.

## Figures and Tables

**Table 1:** Chemical composition of agri-food byproducts (g/100 g) (n = 3).

Parameter	Lupin Hull	Lupin-Byproduct	Camelina Press-Cake
Protein	4.77	14.57	44.54
Fat	0.30	0.59	1.16
Dietary fiber	89.53	74.15	40.30
Ash	2.22	2.85	4.85
Carbohydrates available	3.18	7.84	9.15
Soluble fraction (%)	4.67	4.45	23.58
Insoluble fraction (%)	95.33	95.55	76.42

Data are reported on a dry matter basis. The SD values range from 0 to 5% and are thus regarded as insignificant.

**Table 2:** Composition of Pickering emulsions prior to the spray-drying process.

Formulation	Wall Material		Core Material	
	Agri-Food Byproducts (*w*/*w*, %)	Maltodextrin (*w*/*w*, %)	Oil Phase	Water
1	0.25	19.75	The oil phase prepared at a fixed concentration of 10% (9.8% sunflower oil + 0.2% D-limonene)	70% (phosphate buffer pH 7.0)
2	0.5	19.5		
3	1.0	19.0		
4	2.0	18.0		
5	3.0	17.0		
6	4.0	16.0		

**Table 3:** Effect of type of agri-food byproduct and concentration (0.25, 0.5, 1, 2, 3, and 4%, *w*/*w*) on the moisture, dispersibility, and encapsulation efficiency (EE) of D-limonene powders.

Sample	Concentration (%, *w*/*w*)	Moisture (%)	Dispersibility (%)	EE (%)
Lupin Byproduct	0.25	3.83 ± 0.42 ^BC^	89.61 ± 0.35 ^BC^	42.71 ± 0.73 ^A^
	0.5	3.17 ± 0.31 ^A^	89.74 ± 0.45 ^C^	66.75 ± 1.12 ^B^
	1.0	3.93 ± 0.23 ^C^	90.42 ± 0.38 ^D^	77.33 ± 1.31 ^C^
	2.0	3.10 ± 0.30 ^A^	89.60 ± 0.19 ^BC^	85.44 ± 0.98 ^D^
	3.0	3.43 ± 0.15 ^AB^	89.13 ± 0.15 ^D^	86.49 ± 1.71 ^D^
	4.0	3.43 ± 0.12 ^AB^	85.20 ± 0.25 ^A^	84.97 ± 2.33 ^D^
Lupin Hull	0.25	3.73 ± 0.15 ^AB^	89.48 ± 0.18 ^C^	47.43 ± 1.02 ^A^
	0.5	4.03 ± 0.38 ^B^	90.37 ± 0.18 ^D^	49.20 ± 0.86 ^B^
	1.0	3.93 ± 0.23 ^B^	90.73 ± 0.23 ^D^	60.87 ± 1.50 ^C^
	2.0	3.60 ± 0.14 ^AB^	89.23 ± 0.25 ^BC^	87.62 ± 1.99 ^E^
	3.0	3.50 ± 0.22 ^A^	88.58 ± 0.54 ^AB^	94.33 ± 1.86 ^F^
	4.0	4.47 ± 0.58 ^C^	88.08 ± 0.76 ^A^	76.21 ± 1.61 ^D^
Camelina Press-cake	0.25	3.77 ± 0.40 ^BC^	91.43 ± 0.96 ^AB^	48.91 ± 0.90 ^A^
	0.5	3.50 ± 0.36 ^ABC^	91.12 ± 0.62 ^AB^	58.73 ± 0.89 ^B^
	1.0	3.87 ± 0.15 ^C^	91.71 ± 0.11 ^B^	86.49 ± 2.17 ^E^
	2.0	3.40 ± 0.10 ^AB^	90.84 ± 0.54 ^AB^	80.21 ± 2.08 ^D^
	3.0	3.57 ± 0.12 ^ABC^	90.95 ± 0.28 ^AB^	64.30 ± 1.66 ^C^
	4.0	3.17 ± 0.12 ^A^	90.62 ± 0.16 ^A^	47.88 ± 1.41 ^A^

Different capital letters indicate statistically significant differences (*p* ≤ 0.05) among concentrations of a specific agri-food byproduct. The values represent the mean of three replicates ± SE.

## Data Availability

The original contributions presented in the study are included in the article and Supplementary Material, further inquiries can be directed to the corresponding author.

## Supplementary Materials

The following supporting information can be downloaded at: https://www.mdpi.com/article/10.3390/foods13091326/s1, Figure S1: The pictures correspond to visual characteristics of spray-dried powders obtained from Pickering emulsion stabilized by lupin hull. The spray-dried powders obtained from emulsions stabilized by lupin byproducts and camelina press-cake presented the same characteristics.
