# Supplementary material for: Enhancing the Retention and Oxidative Stability of Volatile Flavors: A Novel Approach Utilizing O/W Pickering Emulsions Based on Agri-Food Byproducts and Spray-Drying
Source: Foods. 2024 Apr 26;13(9):1326. doi: 10.3390/foods13091326 (PMC11083764; doi:10.3390/foods13091326)
Supplement: Supplementary file 1 [file foods-13-01326-s001.zip › foods-2981490-supplementary.pdf]

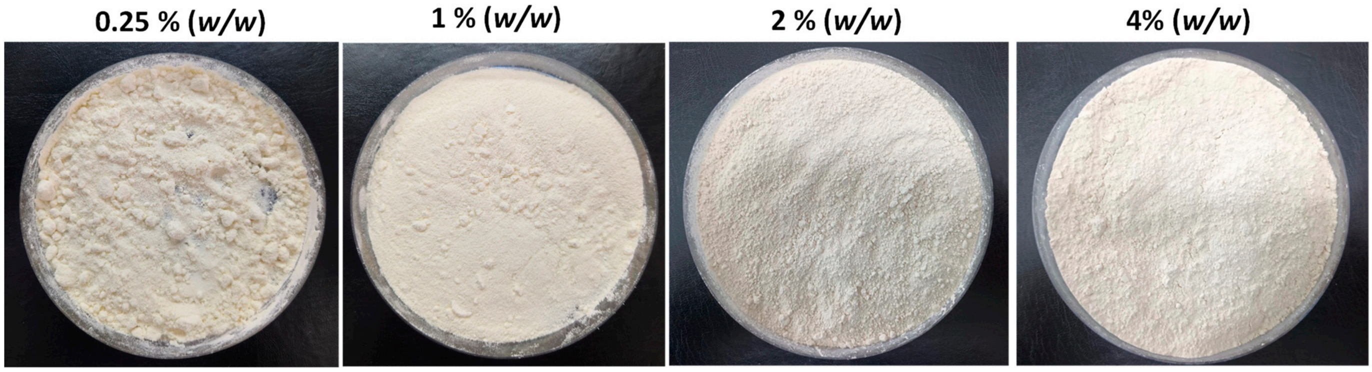

Figure S1. The pictures correspond to visual characteristics of spray-dried powders obtained from Pickering emulsion stabilized by lupin hull. The spray-dried powders obtained from emulsions stabilized by lupin byproducts and camelina press-cake presented the same characteristics.
